# GABA and Gap Junctions in the Development of Synchronized Activity in Human Pluripotent Stem Cell-Derived Neural Networks

**DOI:** 10.3389/fncel.2018.00056

**Published:** 2018-03-06

**Authors:** Meeri Eeva-Liisa Mäkinen, Laura Ylä-Outinen, Susanna Narkilahti

**Affiliations:** NeuroGroup Laboratory, BioMediTech Institute and Faculty of Medicine and Life Sciences, University of Tampere, Tampere, Finland

**Keywords:** human pluripotent stem cells, stem cell derived neurons, microelectrode array, calcium imaging, synchrony, gap junctions, neural network, excitatory GABA

## Abstract

The electrical activity of the brain arises from single neurons communicating with each other. However, how single neurons interact during early development to give rise to neural network activity remains poorly understood. We studied the emergence of synchronous neural activity in human pluripotent stem cell (hPSC)-derived neural networks simultaneously on a single-neuron level and network level. The contribution of gamma-aminobutyric acid (GABA) and gap junctions to the development of synchronous activity in hPSC-derived neural networks was studied with GABA agonist and antagonist and by blocking gap junctional communication, respectively. We characterized the dynamics of the network-wide synchrony in hPSC-derived neural networks with high spatial resolution (calcium imaging) and temporal resolution microelectrode array (MEA). We found that the emergence of synchrony correlates with a decrease in very strong GABA excitation. However, the synchronous network was found to consist of a heterogeneous mixture of synchronously active cells with variable responses to GABA, GABA agonists and gap junction blockers. Furthermore, we show how single-cell distributions give rise to the network effect of GABA, GABA agonists and gap junction blockers. Finally, based on our observations, we suggest that the earliest form of synchronous neuronal activity depends on gap junctions and a decrease in GABA induced depolarization but not on GABA_A_ mediated signaling.

## Introduction

A better understanding of the human brain and its development would allow us to answer questions such as how the brain functions, how the brain malfunctions, and how the brain can be cured. Despite scientific progress, the nature and function of the human brain remains to be fully understood. The brain carries out cognitive functions and behavior using electrical activity. The electrical activity of the brain arises from single neurons communicating with each other. Groups of communicating neurons form neural networks, which function as the computing hubs of the brain. How single neurons give rise to neural network activity remains poorly understood. Patterned neural network activity arises when asynchronous neurons self-organize their firing patterns into synchronous activity (for review Corlew et al., 2004; Blankenship and Feller, 2010; Kerschensteiner, 2014; Luhmann et al., 2016). Different patterns of activity arising during development have been described in several systems and uniformly consist of events of synchronous firing of neurons throughout the neural network separated by periods of silence (Blankenship and Feller, 2010; Moore et al., 2011). These patterns differ in the extent of activity propagation and the fraction of cells recruited (Kerschensteiner, 2014). This synchronous firing has been observed *in vivo* (Landmesser and Szente, 1986; Khazipov et al., 1997) and *ex vivo* (Ben-Ari et al., 1989; Garaschuk et al., 1998; Menendez De La Prida et al., 1998; Corlew et al., 2004), as well as *in vitro* both in primary (Khalilov et al., 1997; Leinekugel et al., 1998) and stem cell-derived cultures (Illes et al., 2007; Heikkilä et al., 2009). However, the series of mechanisms that initiate the events of synchronous network activity and the mechanisms suppressing activity during the quiescent periods are not fully understood (Kerschensteiner, 2014).

The mechanisms responsible for controlling the patterned activity are currently thought to consist of a combination of pacemaker-like membrane properties of single neurons and the network interactions between them (Blankenship and Feller, 2010; Momose-Sato and Sato, 2013; Kerschensteiner, 2014). The network interactions mediated by traditional synaptic neurotransmitters in developing neural networks have been shown to work also via extra synaptic transmission (Blankenship and Feller, 2010). Additionally, the mechanisms act homeostatically, adapting to induced impairments by employing an alternative mechanism (Blankenship and Feller, 2010; Kerschensteiner, 2014). However, patterned activity has also been shown to arise in *in vitro* cell cultures, which provide a simpler system for studying its basic mechanisms (Opitz et al., 2002; Sun et al., 2010). Despite the complexity of the environment during development, the network interactions that participate in the patterning of activity during synchronous network events have been found to include a traditional synaptic neurotransmitter gamma-aminobutyric acid (GABA) and gap junctions (Momose-Sato and Sato, 2013). GABA is an inhibitory synaptic neurotransmitter of the adult brain, but in the immature brain, GABA can also excite neurons (Feller, 1999; O’Donovan, 1999; Ben-Ari, 2002). Similarly, gap junctional coupling in an adult brain differs from that of an immature one (Blankenship and Feller, 2010). The role of both GABA and gap junctions in driving the patterned network activity remains uncertain as the reports from different *in vivo* and *ex vivo* preparations contradict each other (Conhaim et al., 2011; Kerschensteiner, 2014). Furthermore, species differences in preparations might also interfere in the translatability across species, which has been shown to be especially poor in the brain (Haston and Finkbeiner, 2016).

Several aspects differ between the primate and the rodent brain (Finlay and Darlington, 1995). For example, developing primate neural networks contain primate-specific stem cell populations and neurons (Hill and Walsh, 2005; Rakic, 2009). The cellular constituents of human-specific neural development can be successfully captured in human pluripotent stem cell (hPSC)-derived neural cultures (Shi et al., 2012). Here, we studied the contribution of GABA and gap junctions to the development of synchronous activity in hPSC-derived neural networks. Developing networks were measured simultaneously at a single neuron resolution with calcium imaging and on a network level with microelectrode arrays (MEAs). The single-neuron responses were associated with the network responses by comparing the calcium imaging and MEA recordings as well as by employing a large scale single-cell analysis. In this article, we show that the strength of GABA excitation played a key role in the formation of network-wide synchrony. Furthermore, synchronous network activity was produced by neurons of different functional maturity levels acting in concert. Finally, we show that the synchronous network activity in hPSC-derived neural networks was not mediated by endogenous GABA and is modulated by gap junctions.

## Materials and Methods

### Human Embryonic Stem Cells

The neural networks used in this study were differentiated from the human embryonic stem cell line Regea 08/023 (Regea 08/023, European Human Embryonic Stem Cell Registry). Regea 08/023 has been previously derived (Skottman, 2010) and was maintained as previously described (Toivonen et al., 2013). This work was conducted under the approval of the Ethics Committee of Pirkanmaa Hospital District for the derivation, characterization, and differentiation of hESC-lines (Skottman, R05116) and under an approval of Valvira, the Finnish National Authority for Medicolegal Affairs, for research on human stem cells (1426/32/300/05).

### Neural Cell Derivation, Cell Plating and Coating

The neural differentiation of hESCs was performed as described by Lappalainen et al. (2010). Basic fibroblast growth factor (bFGF) was used at 20 ng/ml during the neurosphere phase. After suspension differentiation, the neurospheres were mechanically dissected.

**Figure 1:**
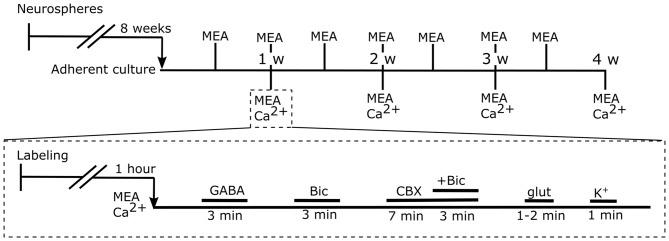
Timeline for the experiments. Top timeline: human pluripotent stem cell (hPSC)-derived neural precursors were cultured as neurospheres for 8 weeks. Next, neural precursors were plated and cultured as adherent cells in order to form neural networks. The functional maturation of the networks was followed by microelectrode array (MEA) measurements twice a week. At each of the 4 weeks, an end-point measurement, MEA and Ca^2+^, was performed for 5–9 neural cultures (Table 1, column 1). Bottom timeline: first, neural cells were labeled for calcium imaging. Next, simultaneous MEA and Ca^2+^ recording was performed, and a series of pharmacological agents were applied. The series consisted of gamma-aminobutyric acid (GABA), the GABAA receptor antagonist bicuculline (Bic), the gap junction blocker carbenoxolone (CBX), glutamate (glut) and high potassium (K+).

The pieces of aggregates were plated on thin MEA dishes with a 180-μm-thin recording area, 59 substrate-embedded titanium nitride microelectrodes, a silicon nitride isolator, and an internal reference electrode (60ThinMEA200/30iR-ITO, Multi Channel Systems MCS GmbH). Electrodes with a 30-μm diameter were organized in an 8 × 8 grid with 200 μm spacing. MEA dishes were coated (overnight at +4°C or 2 h at +37°C) with PEI (0.1% polyethylenimine, Sigma-Aldrich) and subsequently with mouse laminin (20 μg/ml, Sigma-Aldrich, St. Louis, MO, USA). Altogether, 36 (29/36 were successful) neural networks were plated onto MEAs. The bFGF was withdrawn at the beginning of adherent culture in MEA. Half of the culture medium was replaced three times a week. The timeline for the experiments is shown in Figure 1.

### Follow-Up Recordings

MEA follow-up measurements were performed twice a week for 4 weeks or until the end-point measurement. The follow up measurements were performed with the MEA wells sealed with semi-permeable membranes (ALA MEA-MEM, ALA Scientific Instruments, Westbury, NY, USA). The voltage from the microelectrodes was measured with a filter amplifier MEA2100 (Multi Channel Systems MCS GmbH, Reutlingen, Germany). The temperature was controlled with an external heater unit (TC02, MCS) set to +38°C, and the cultures were allowed to settle for 1 min in the system before each 5-min follow-up recording. An analog-to-digital conversion was performed at a 50-kHz sampling frequency. The same recording setup was used during combined MEA recording and calcium imaging except the semi-permeable membrane was not used.

### End-Point Recordings

#### Preparation for Calcium Imaging

For the end-point measurement, neural cells (Table 1, 1st column) were loaded with Fluo-4 AM (4 μM, F14201, Thermo Fisher Scientific) diluted in fresh cell culture medium for 30 min at 37°C and 5% CO_2_. This was followed by a wash with fresh Ringer’s solution for 30 min at 37°C and 5% CO_2_. The Ringer’s solution contained 140 mM NaCl, 10 mM HEPES, 10 mM D glucose, 3.5 mM KCl, 1.25 mM NaH_2_PO_4_, 2 mM CaCl_2_ and 1 mM MgCl_2_ (all from Sigma-Aldrich) dissolved in dH_2_O, pH adjusted to 7.4 with NaOH.

**Table 1:** Numbers of measured and analyzed samples.

Age at endpoint	Calcium imaged (& MEA measured) networks	Analyzed calcium imaged networks (on MEA)	Analyzed ROIs (networks)	Analyzed synchronous networks (ROIs)
1 w	7 (5)	5 (4)	3496 (5)	-
2 w	13 (6)	4 (3)	2132 (4)	-
3 w	11 (7)	4 (3)	2136 (4)	3 (1358)
4 w	11 (9)	4 (4)*	2018 (3)	2 (1216)
Total	42 (27)	17 (14)	9782 (16)	5 (2574)

All the networks measured with MEAs were simultaneously calcium imaged. Likewise, the calcium imaging was analyzed from all the networks whose MEA activity was analyzed. *One of the networks was excluded from detailed quantification (Analyzed ROIs) due to the amount of noise in the recording. w refers to week, MEA refers to microelectrode array, ROI refers to region of interest, in this case, individual neuron.

#### Pharmacology

In the beginning of each combined MEA recording and calcium imaging session (Table 1, 1st column), a baseline was measured after the culture was perfused with Ringer’s solution for at least 10 min. Pharmacological substances were applied to neural cultures via a gravitation-fed perfusion system (2 ml/min, ALA Scientific). Before the cells were perfused, the substances were diluted to their final concentrations. Treatments were applied in the following order: GABA (100 μM, GABA, A5835-10 g, Sigma), GABA_A_ receptor antagonist bicucullinemethionine (10 μM, Bic, 14343-10MG, Sigma-Aldrich), gap junction blocker carbenoxolone (CBX; 25 μM, CBX, C4790, Sigma-Aldrich), L-glutamic acid (30 μM, glut, G8415-100 g, Sigma), and KCl (5 mM, K+, P9541-500G, Sigma-Aldrich). Each substance application was separated by a 3–5-min perfusion with Ringer’s solution.

#### Calcium Imaging

During calcium imaging, the neural network activity was imaged every 0.5 s (2 Hz) with a fluorescent imaging system consisting of an Olympus IX61 inverted microscope (10× objective, NA = 0.5), an Andor iXon 885 EMCCD camera (Andor Technology, Belfast, Northern Ireland) and a Polychrom V monochromator (TILL Photonics, Munich, Germany). Images were acquired with TILL Photonics Live Acquisition software. Images were exported into TIFF-stacks from TILL Photonics Offline Analysis software and loaded into MATLAB.

### MEA Analysis

Altogether, recordings from 14 neural networks (3–4 per timepoint, Table 1, 2nd column), all also included in the calcium analysis, were analyzed (Supplementary Figure S1). The digitized recordings were imported to MATLAB (MathWorks, Inc., Natick, MA, USA) using the NeuroShare library (NeuroShare Library, 2003). The recorded raw MEA signals were noisy due to the noise caused by the perfusion and imaging system. Noise was removed by subtracting the median voltage of all electrodes from the voltage of each individual electrode. The signal was bandpass filtered (200–3000 Hz). Several windows (0.05, 0.1, 0.5, 1, 10, 30, 50, 70, 90, 100, 300 s) with different overlap (30%, 50% and 80%) were compared for optimal noise reduction. Finally, the signal was analyzed in 30-s windows with an 80% overlap. The median of window noise was calculated, and spikes were detected as events that crossed five times the median threshold similar to Quiroga et al. (2004). Detector dead time was 1.5 ms. Spike timestamps and cut-out waveforms (spanning 1 ms prior and 2.2 ms after the maximum of the spike) were stored.

### Quantification and Presentation of MEA Data

**Figure 2:**
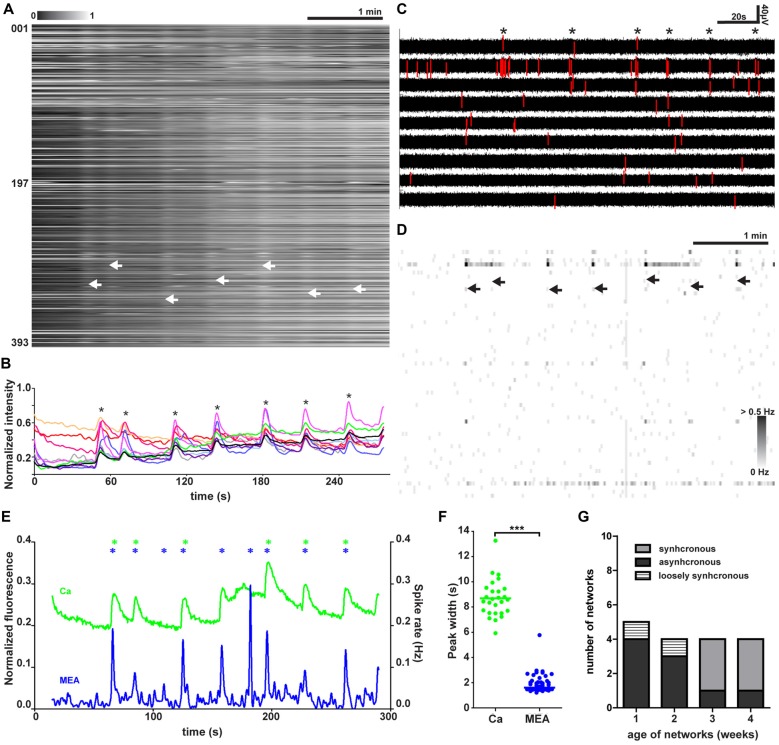
Spontaneous synchronized network activity during baseline. **(A)** Normalized raster plot of spontaneous intracellular Ca^2+^ fluctuations from all neurons (*n* = 393) in the imaged area from one synchronous network. Each row of pixels (y-axis, number of neuron) contains normalized intensity changes (gray scale bar) during a 5-min (x-axis) recording from one neuron. Vertical stripes (arrow heads) denote network-wide synchronous intracellular calcium rises. **(B)** Representative single-neuron intracellular calcium level traces (y-axis) from 10 representative synchronously active neurons. Neurons are selected from the recording shown in **(A)**. Traces show the normalized fluorescent calcium dye intensity (y-axis) over time (x-axis). The asterisks mark synchronous intracellular calcium rises. **(C)** MEA recording from nine selected electrodes during baseline activity in one network. Each row contains a voltage trace from one electrode. Asterisks mark the moments of synchronous activity. Voltage values associated with detected spikes are drawn in red. Vertical scale bar denotes a 40-μV change in the measured voltage, and the horizontal scale bar denotes 20 s of time. **(D)** MEA recording as a grayscale spike rate raster plot during synchronous baseline activity from the same network at the same time as in **(A)**. Spikes detected from individual electrodes are indicated by gray levels according to the associated scale bar. Each row represents the spike rate (gray scale bar) detected by one electrode (*n* = 60, y-axis) on one row in the grayscale raster plot. Synchronous network activity marked with arrow heads. **(E)** Network activity as summed Ca^2+^ fluctuations (green) and global MEA firing rate (blue) from one representative network (393 neurons) during baseline. Vertical scale bar corresponds to a 20% change in fluorescence (green trace) and 0.02-Hz change in spike rate (blue trace). Asterisks mark the peaks of network activity detected during the baseline for the summed calcium signal (green) and whole MEA spike rate (blue). **(F)** Widths of the network activity peaks from summed Ca^2+^ fluctuations (green, *n* = 27 peaks) and global MEA firing rate (blue, *n* = 46 peaks) for all networks (*n* = 5 networks). ***marks significant (*p* < 0.001) difference between peak widths measured by calcium imaging or MEA during baseline.** (G)** Number and relative proportion of synchronous, asynchronous and loosely synchronous network activity found at each time point.

A grayscale spike rate raster plot was obtained by calculating the spike rate from the spike timestamps for each electrode with 2-s binning. The spike rate was then represented as a grayscale from white to black [0, 0.5 Hz]. For network-level analysis, a combined spike rate (10-s bins, 80% overlap) from all electrodes, with the exception of the ground electrode, was calculated. Network-wide peaks in spike rate were detected using the MATLAB Signal Processing Toolbox. The prominence of an activity peak was calculated as the difference in activity between the peak and the surrounding baseline. Thus, the prominence allowed for the comparison of the increase in activity whether the baseline contained a high or low amount of activity. A prominence of 0.05 was used as a criterion for activity peaks. Peak detection provided values for peak width, prominence, height and timing. The values are represented in images per peak (dots in Figure 2F) and as medians of detected peaks (horizontal lines in Figure 2F).

### Calcium Imaging Analysis

To extract information from all of the cells in the imaged area, an automated segmentation was performed in MATLAB. Altogether, recordings from 17 neural networks (we randomly selected a set of measurements to reach 4–5 measurements per timepoint, Table 1, 2nd column) were analyzed. All of the imaging recordings corresponding to the analyzed MEA recordings were analyzed.

### Segmentation

In MATLAB, images taken during calcium imaging were segmented with a foreground and background marker-guided watershed transformation (Supplementary Figure S2). Images were preprocessed in MATLAB by averaging 50 subsequent frames and scaling pixel values to range [0, 1].

Foreground markers were generated by a sequence of morphological operations. A threshold marker was used to identify high-intensity objects when performing morphological opening on the preprocessed image. The result of the morphological opening was subtracted from the averaged and scaled image. The resulting image was eroded with a flat morphological disk-shaped structuring element with a radius of 2. Next, the regional maxims were detected from the processed image, and the result was first eroded and then dilated by using a 3 by 3 neighborhood for both operations.

Background markers were generated by setting the value of the pixels in the foreground marker locations on the preprocessed image to the same value. The image was converted to a black-and-white image by thresholding with Otsu’s (1979) method. The resulting binary image was used to compute the Euclidean distance transform further used in the Fernand Meyer watershed algorithm (Meyer, 1994). The watershed regions were set to 0, thus leaving the outside areas background markers with a value of 1. To ensure that none of the foreground and background markers were touching, the background markers were dilated with a flat morphological disk-shaped structuring element with a radius of 2, and the resulting pixels were removed from the set of foreground pixels.

For the final watershed transformation, the preprocessed image was parallel filtered with a predefined Sobel horizontal edge-emphasizing 2D-filter and its transpose. The values outside the bounds were assumed to be equal to the array border value for both operations. A watershed transformation was performed on an image generated from the square root of the sum of the exponents of the filtering operations with regional minimums imposed to locations specified by foreground and background markers.

### Tracking

The segmentation results were tracked by minimizing the distance between the centroids of detected cells. This was performed by using a cost matrix generated from the distances between the centroids of areas resulting from segmentation. A gating threshold of 20 was used for distances together with a gating cost of 5000. The cost of non-assignment was set to 500. The minimum track age was set to 20 and visibility to 0.5 of the time. The optimal track was calculated with the James Munkres’s variant of the Hungarian assignment algorithm (Munkres, 1957).

The track information was used to generate a fluorescence intensity trace for each cell. Altogether, traces from 9782 cells were collected. Each fluorescent intensity trace was normalized to [0, 1]. Thus, the intensity value 1 reflects the maximum intensity of a cell during the recording, and measured responses are expressed as a fraction of the saturation level of the fluorescent signal (Carmignoto et al., 1998; Maravall et al., 2000).

The collected traces were filtered with a Butterworth bandpass filter (0.0013–0.02 Hz), and traces with a standard deviation greater than 0.05 were removed as noisy. Traces with less than a 10% increase in normalized fluorescence during the application of high potassium were removed as inactive or originating from cells other than neurons. The amount of remaining traces in a network was always more than 98% of the initial traces.

#### Quantification and Presentation of Calcium Imaging Data

The images of all included calcium traces (117–1354 traces per image) were formed by aligning all individual calcium traces of a recording (*n* = 17 networks, Table 1, 2nd column). To estimate the network response (*n* = 5 networks), the individual traces were bandpass filtered [0.005–0.2 Hz] with a Butterworth filter and normalized. The traces were then summed, and the sum was normalized. Network activity peaks were detected when the peak prominence exceeded 0.025. The same criterion was used for individual traces (*n* = 2574 traces from synchronous networks). Peak detection for both individual traces and network traces was done with the MATLAB Signal Processing Toolbox. Peak detection provided values for peak width, prominence, height and timing. The values are represented in result images per peak (dots in Figures 2F, 5C, 6D, 7D,E) and as medians of detected peaks (asterisks in Figures 5E,F, 6F, 7F,G). The change in peak prominence or inter-peak interval was counted as difference from baseline. Relative change was obtained by dividing the change with the baseline value. The distribution of peak prominences and inter-peak intervals were represented as histograms obtained with 0.005 and 1-s binning, respectively. Change distributions were also represented as a histogram, all with 5% binning. Cumulative sums were obtained from the aforementioned histograms. Excitatory GABA responses were calculated by low-pass filtering traces with a 0.024-Hz Butterworth filter and summed during baseline and GABA application. The difference between these two was taken as the excitatory GABA response.

**Figure 3:**
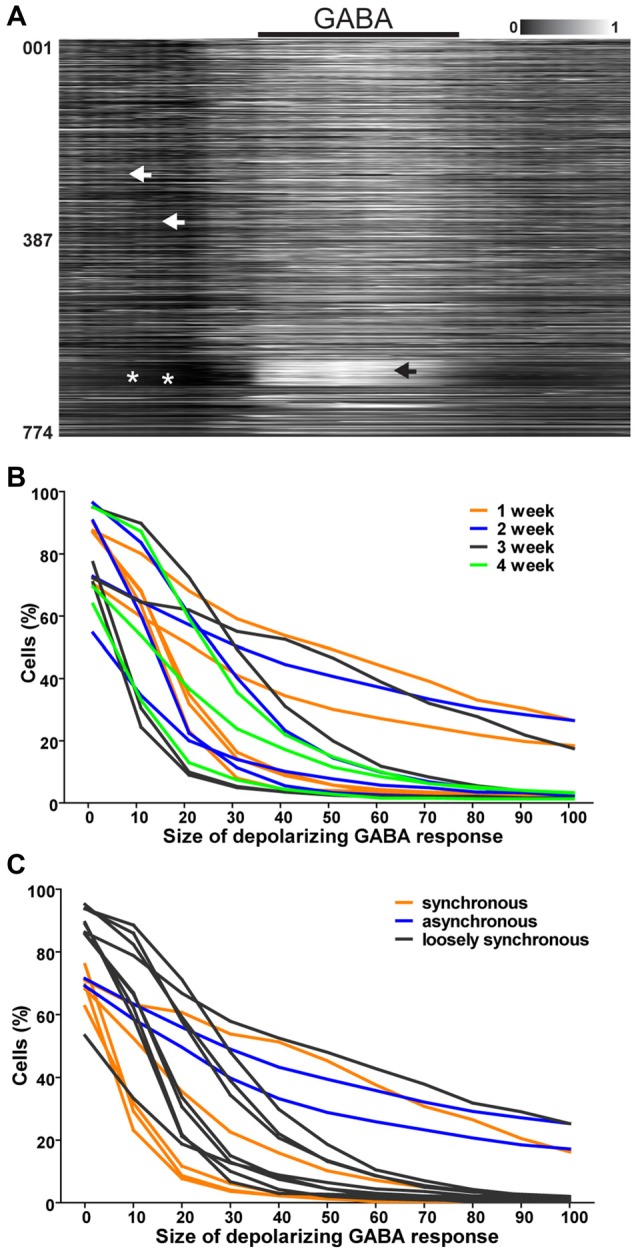
GABA responses in single neurons. **(A)** Normalized raster plot of spontaneous intracellular Ca^2+^ fluctuations from all neurons (*n* = 774) in the imaged area from one loosely synchronous network during GABA application (black bar, 3 min). Each row of pixels (y-axis, number of neuron) contains normalized intensity (gray scale bar) changes during a 10-min (x-axis) recording from one neuron. Black arrow head points to a group of neurons with a high intracellular calcium rise in response to GABA application. White arrow heads point to events of loose synchronous activity. White asterisks mark the lack of synchrony. **(B)** Reverse cumulative sum of % of neurons in the field of view (y-axis) with respect to the depolarizing response of single neurons to GABA application (x-axis) is shown for each network. Neurons from 1 (*n* = 774, 1354, 472, 493 and 403), 2 (*n* = 645, 454, 538 and 495), 3 (778, 117, 670 and 571) and 4 (*n* = 585, 823, 393) weeks old networks are represented with orange, blue, black and green respectively. **(C)** Reverse cumulative sum of % of neurons in the field of view (y-axis) with respect to the depolarizing response of single neurons to GABA application (x-axis) is shown for each network. Neurons from networks with synchronous activity (*n* = 117, 670, 571, 823 and 393 neurons), no synchrony (*n* = 585, 778, 454, 1354, 493, 495, 472, 403 and 531 neurons) and loose synchrony (*n* = 774 and 645 neurons) are represented with orange, blue and black, respectively.

**Figure 4:**
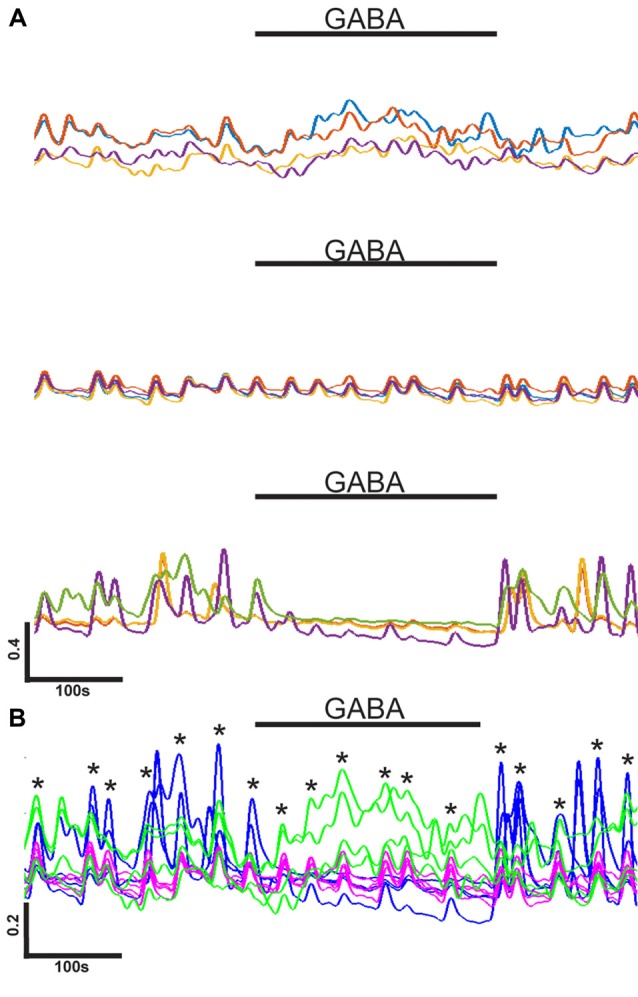
Heterogeneous GABA responses in single network. **(A)** Groups of four representative normalized calcium level (y-axis) traces with different responses to GABA application (black bars) within one network. Traces are examples of a slight increase in intracellular calcium (top), no response (middle), and an inhibition of activity (bottom) during GABA application. Horizontal scale bar corresponds to 100 s. Vertical scale bar corresponds to a 0.4 (40% of max) increase in calcium indicator fluorescence intensity. **(B)** Example of synchrony between differently behaving neurons in the same network [same as in **(A)**]. Traces are from four neurons exited by GABA (green), four neurons not responding to GABA (purple/pink), and four neurons inhibited by GABA (blue). Black bar corresponds to GABA application. Asterisks mark the moments on synchronous activity peaks. Horizontal scale bar corresponds to 100 s. Vertical scale bar corresponds to a 0.2 (20% of max) increase in calcium indicator fluorescence intensity.

**Figure 5:**
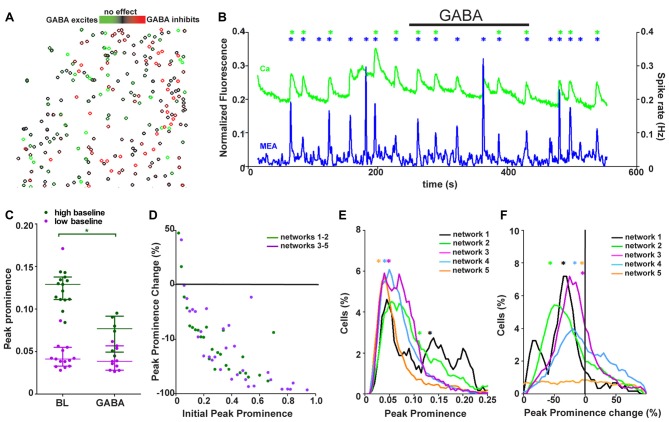
Synchronously active networks are a heterogeneous mixture of synchronously active neurons with different GABA responses. **(A)** Spatial distribution of neurons (*n* = 393) colored based on their response to GABA (from green to black = strongly excited to not responding, from black to red = not responding to strongly inhibited) from one representative network. **(B)** Network activity as summed Ca^2+^ fluctuations (green, 393 neurons) and global MEA firing rate (blue) from one representative network during GABA application (back bar, 4 min). Black bar shows the time of GABA application. Asterisks mark the population activity peaks detected from the network activity traces (calcium imaging = green, MEA = blue). **(C)** Peak prominence of Ca^2+^ network activity [example trace represented in green in **(B)**]. Each dot represents an individual peak prominence (marked with asterisks in **B**) of network activity during baseline and GABA application. Peaks of activity from different groups of networks are represented by different colors. Horizontal bars represent the median network activity peak prominence for each network group. *marks significant (*p* < 0.05) difference between peak prominence during baseline and GABA application. **(D)** The single-neuron responses to GABA as a change in peak prominence (y-axis) plotted against the initial peak prominence (x-axis) from 688 (dark green, neurons from networks with strong GABA inhibition) and 1886 (purple, neurons from networks with barely any GABA response) neurons from the synchronous networks. **(E)** Distributions of the single-neuron peak prominence during baseline for all synchronous networks. Different networks are represented by different colors (black: 117, orange: 670, green: 571, blue: 823 and purple: 393 neurons). Distribution depicts the peak prominence (x-axis) and the % of neurons in the network with the corresponding peak prominence (y-axis). The asterisks represent the medians of the peak prominence of the network activity. **(F)** Distributions of single-neuron peak prominence changes in response to GABA application for all synchronous networks. Different networks are represented by different colors (black: 117, orange: 670, green: 571, blue: 823, and purple: 393 neurons). Distribution depicts the peak prominence % change (x-axis) and the % of neurons in the network with the corresponding peak prominence change (y-axis). The asterisks represent the medians of the peak prominence change in the network activity.

**Figure 6:**
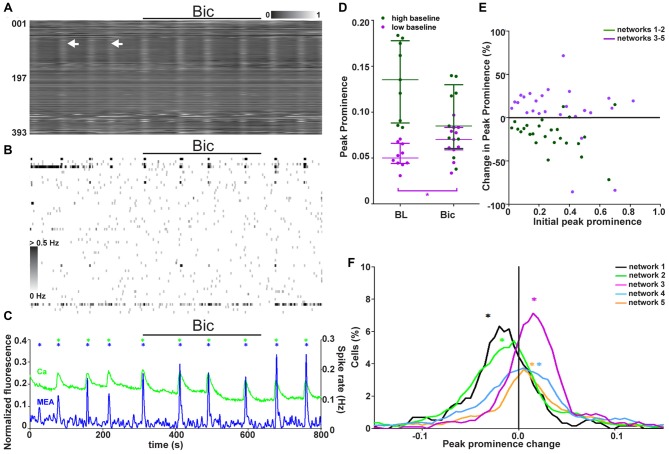
Two classes of networks with different GABAergic signaling. **(A)** Normalized raster plot of spontaneous intracellular Ca^2+^ fluctuations from all neurons (*n* = 393) in the imaged area from one synchronous network during GABAA receptor blocker (bicuculline (Bic), 3 min) application (black bar). Each row of pixels (y-axis, number of neuron) contains the normalized intensity (gray scale bar) changes during a 7-min (x-axis) recording from one neuron. Vertical stripes (arrow heads) denote synchronous intracellular calcium rises. **(B)** A grayscale spike rate raster plot of an MEA recording from the same network at the same time as calcium imaging in **(A)**. Spikes detected from individual electrodes (*n* = 60, y-axis) are indicated by gray levels according to the associated scale bar. Black bar denotes GABA receptor blocker (Bic) application. Timescale (x-axis) is the same as in **(A)**. **(C)** Network activity as summed Ca^2+^ fluctuations (green) and global MEA firing rate (blue) from one representative network (393 neurons, same as in **A,B**) during GABAA receptor blocker (Bic) application (back bar). Asterisks mark the population activity peaks detected from the network activity traces (calcium imaging = green, MEA = blue). **(D)** Peak prominence of the Ca^2+^ level-based network activity (example trace represented green in **C**). Each dot represents an individual peak prominence (marked with asterisks in **C**) of network activity during baseline and GABAA receptor blocker (Bic) application. Peaks of activity from different groups of networks are represented by different colors. Horizontal bars represent the median network activity peak prominence for each network group. *marks significant (*p* < 0.05) difference between peak prominence during baseline and blocker application.** (E)** The single-neuron responses to GABA_A_ receptor blockade as a change in the peak prominence (y-axis) plotted against the initial peak prominence (x-axis) from 688 (dark green, neurons from networks with high initial activity) and 1886 (purple, neurons from networks with low initial activity) neurons from the synchronous networks.** (F)** Distributions of single-neuron peak prominence changes in response to GABA_A_ receptor blockade for all synchronous networks. Different networks are represented by different colors (black: 117, orange: 670, green: 571, blue: 823, and purple: 393 neurons). The distribution depicts the peak prominence % change (x-axis) and the % of neurons in the network with the corresponding change (y-axis). The asterisks represent the medians of the peak prominence changes in the network activity.

**Figure 7:**
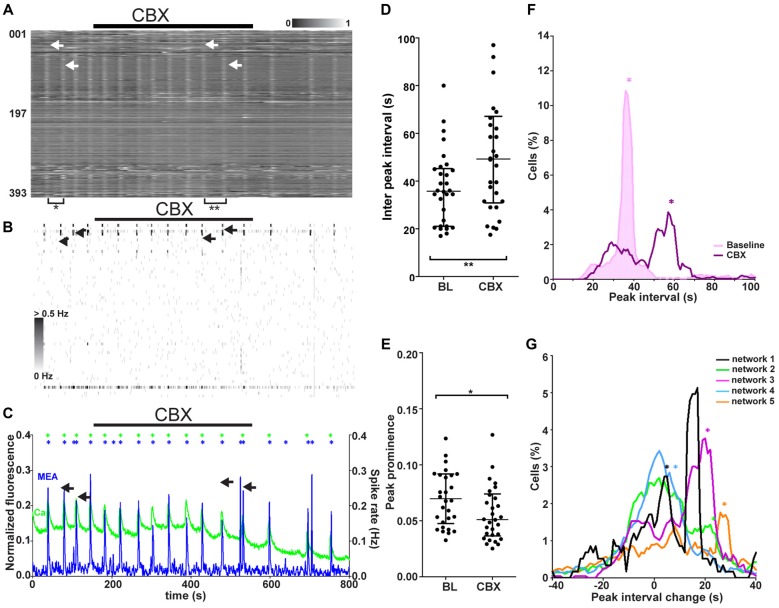
Network responses to blockade of gap junction-mediated signaling in synchronous networks. **(A)** Normalized raster plot of spontaneous intracellular Ca^2+^ fluctuations recorded from all neurons (*n* = 393) in the imaged area from one synchronous network during baseline (BL) and gap junction blocker (CBX) application (black bar). Each row of pixels (y-axis, number of neuron) contains the normalized intensity (gray scale bar) changes during a 14.5-min (x-axis) recording from one neuron. Vertical stripes (arrow heads) denote synchronous intracellular calcium rises. Brackets mark the gap between vertical lines of synchronous activity, the inter-peak interval, during baseline (*) and CBX application (**). The arrows indicate two adjacent network events, one during baseline and one during CBX application. **(B)** A grayscale spike rate raster plot of an MEA recording from the same network at the same time as the calcium imaging in **(A)**. Spikes detected from individual electrodes (*n* = 60, y-axis) are indicated by gray levels according to the associated scale bar. Black bar denotes gap junction blocker (CBX) application. Timescale (x-axis) same as in **(A)**. The arrows indicate two adjacent network events, one during baseline and one during CBX application. **(C)** Network activity as summed Ca^2+^ fluctuations (green) and global MEA firing rate (blue) from one representative network (393 neurons, same as in **A,B**) during gap junction blocker (CBX) application (back bar). Asterisks mark the population activity peaks detected from the network activity traces (calcium imaging = green, MEA = blue). Duration of the shown signals is the same as in **(A)**. The arrows indicate two adjacent network events, one during baseline and one during CBX application. **(D)** Inter-peak interval of Ca^2+^ level-based network activity (example trace represented green in **C**). Each dot represents an individual value between two adjacent peaks (marked with asterisks in **C**) of network activity during baseline or gap junction blocker (CBX) application (3 min during the end of application). Horizontal bars represent the median network activity peak intervals. **marks significant (*p* < 0.01) difference between inter-peak interval during baseline and blocker application.** (E)** Peak prominence of Ca^2+^ level-based network activity (example trace represented green in **C**). Each dot represents an individual peak prominence (marked with asterisks in **C**) of network activity during BL and gap junction blocker (CBX) application. Only the last 3 min of activity during CBX application were taken into consideration due to the slow effect of CBX. Horizontal bars represent the median network activity peak prominence. *marks significant (*p* < 0.05) difference between peak prominence during baseline and blocker application.** (F)** Single-neuron (*n* = 393) inter-peak interval distribution of one representative network during baseline (pink shaded area) and gap junction blocker application (purple non-shaded area). The distribution depicts the inter-peak interval (x-axis) and the % of neurons in the network with the corresponding interval (y-axis). The asterisks (now vertical lines) represent the inter-peak interval of the network activity (same as horizontal lines in **D**) during baseline (pink) and gap junction blockade (purple). **(G)** Distributions of single-neuron inter-peak interval changes in response to gap junction signaling blockade for all synchronous networks. Different networks are represented by different colors (black: 117, orange: 670, green: 571, blue: 823, and purple: 393 neurons). The distribution depicts the inter-peak interval % change (x-axis) and the % of neurons in the network with the corresponding change (y-axis). The asterisks represent the medians of the inter-peak interval changes in the network activity.

#### Statistical Analysis

For data represented in Figures 2F, 5C, 6D, 7D,E Wilcoxon signed rank test (paired, two-tailed, nonparametric) was performed in GrapPad Prim 5.02. For data represented in Figures 5D, 6E the Pearson correlation coefficients and *p*-values were calculated in MATLAB.

## Results

### Emergence and Properties of Synchronous Network Activity

To study the development of neuronal network functionality, hPSC-derived neural cells were plated on top of MEAs and allowed to spontaneously form neural networks (*n* = 17, Supplementary Figure S3). The development of activity in neural cell cultures was followed by measuring extracellular voltage signals twice a week with the underlying MEA. In addition, at 1-week, 2-week, 3-week, and 4-week, and 4-w time points, intracellular calcium level changes were measured simultaneously with MEA recording (Table 1, column 1). During this measurement, a series of pharmacological agents (see “Materials and Methods” section Figure 1) was applied.

Live-cell calcium imaging during baseline revealed three distinct patterns of activity. We labeled these patterns of activity as synchronous, asynchronous and loosely synchronous activity. A representative recording from one synchronous network is shown in Figure 2A. The synchronous activity was marked by tightly synchronous rises of intracellular calcium seen as white vertical stripes with clear borders when measurements from all neurons in the imaged field of view were aligned. A representative recording from one asynchronous network is shown in Supplementary Figure S4C. The aligned traces from asynchronous networks did not show any stripes. A representative recording from one loosely synchronous network is shown in Supplementary Figure S4D. Loosely synchronous networks were distinguished from asynchronous by the presence of stripes, but when compared to synchronous networks their borders were not clear-cut and contained staggered ends of calcium traces. The difference between synchronous and loosely synchronous networks was more clear when comparing individual neuronal traces (Figure 2B and Supplementary Figure S4B, respectively). In synchronous networks the neurons were mostly silent between network events and had their highest activity during the event, while the activity during events was not remarkably different from the activity between network events in the loosely synchronous networks.

These synchronous network events occurred at a median rate of 0.03 Hz (~2 per minute, *n* = 5 networks, Table 1, column 4), but the interval between events varied on average from 18 s to 49 s. The activity was studied more closely at the level of a single trace, and the activity of 10 individual neurons from a synchronous network is shown in Figure 2B. From single-neuron traces, it was possible to see that the participating neurons were not active during every network event and that the level of increase in calcium in a cell during synchronous activity varied between events.

Synchronized activity was also detected in the simultaneously performed MEA recordings (*n* = 5, Table 1, column 4). On MEA recordings, the synchronous network events were seen as an increase in the number of detected spikes occurring in a limited time window across several electrodes. Figure 2C shows a representative recording from selected electrodes from one network. When the spikes trains were observed more closely, the number of detected spikes associated with network events clearly varied between events. Furthermore, few spikes were also detected during the less active intervals between the network events. Electrodes measuring the highest levels of activity within one network were usually participating in the synchronous activity as well. Spike rates across the MEA during several network events in one neural culture are shown in Figure 2D.

Next, the network responses of the calcium imaging and MEA recordings were formed by summing the signal of individual components, single-neuron calcium traces or single-electrode spike rates, respectively (*n* = 5, Table 1, column 4). Aligned network responses from calcium imaging and the MEA recording from one representative network are shown in Figure 2E. The intracellular calcium rises and the fast extracellular field potential changes on MEA were temporally associated with each other. The temporal association between the calcium imaging and MEA recording indicates that the recorded intracellular calcium rises arise from action potential firing. However, one of five networks with synchronous activity associated with the intracellular calcium rises did not show any synchronous activity on the MEA recording. Compared to the intracellular calcium rises, the extracellular signals recorded during the synchronous activity peaked higher above baseline and returned back to baseline faster. This was further studied by quantifying the width of network activity peaks. The widths of network activity peaks of network events are shown in Figure 2F. The action potential firing window (1.6 s, median of 27 peaks from five synchronous networks) was significantly (*p* < 0.001) smaller and more stable than the window of intracellular calcium fluctuation (8.7 s, median of 46 peaks from five synchronous networks).

The synchronous activity, seen as stripes in aligned Ca^2+^ traces, was detected in neural networks earliest after 3 weeks of adherent culture. The amount of different types of network activity patterns at each week of adherent culture is shown in Figure 2G (*n* = 17, Table 1, 2nd column). Before 3 weeks, network activity was mainly asynchronous (78%, 7 of 9, Supplementary Figure S4), with a minority (22%, 2 of 9) of networks loosely synchronous (Supplementary Figure S4). After 3 weeks, the majority (80%, 6 of 8) of the recorded networks manifested synchronous activity, with a minority of asynchronous networks (20%, 2 of 8).

To summarize, we observed the emergence of synchronous activity in hPSC-derived neural networks earliest at 3 weeks. The synchronous activity was observed as population-wide increases in activity measured simultaneously by calcium imaging and MEA.

### GABA Response in Single Cells

The responses to GABA application during combined calcium imaging and MEA recording were analyzed. The assessment of aligned intensities from whole networks (*n* = 17) revealed that loosely synchronized neural networks (*n* = 2 networks, *n* = 1419 neurons) contained strongly GABA-depolarized neurons that did not participate in synchronous network events. A representative recording during GABA application from a loosely synchronous network is shown in Figure 3A.

The role of the depolarizing GABA response of individual neurons in relation to the generation of a synchronously active network was further studied by quantifying the single-neuron responses (*n* = 8959, Table 1, column 3) to GABA application. There was no clear connection between the network age and response to GABA. Thus, we formed three groups based on the network activity pattern (synchronous, loosely synchronous, and asynchronous). The proportions of neurons within each network with respect to the GABA response level are shown in Figures 3B,C. The stronger the GABA depolarization, the smaller the proportion of those responses in synchronous networks compared to the proportion in asynchronous and loosely synchronous networks. No clear change in the network composition was observed regarding the culture age. Thus, synchronous networks were the least GABA depolarized when compared to asynchronous and loosely synchronous networks. Furthermore, each of the synchronous networks (5 of 5) had a distribution of GABA responses from depolarizing to inhibitory.

Individual traces were compared more closely to study their participation in the network-wide synchronous activity. Four representative traces from three types of observed responses are shown in Figure 4A. The individual neurons within one network could, for example, be moderately depolarized, have no response or be inhibited as a response to GABA application. Traces representing different responses were combined to study if there was synchrony between neurons with different responses to GABA. A representative alignment is shown in Figure 4B. Neuronal cells with different GABA responses were observed to participate in the synchronous activity together within the network (5 of 5).

In summary, we observed that the presence of synchronous spontaneous activity in hPSC-derived networks was associated with a lack of strongly GABA-depolarized neurons and a smaller proportion of neurons depolarized by GABA than in asynchronous and loosely synchronous networks. Furthermore, we observed that strongly GABA-depolarized neurons are often not participating in network activity patterns.

### GABA Response in Synchronously Active Networks on the Network Level

To reveal any spatial organization of neurons within the networks with respect to GABA responses, the neurons of the imaged area were pseudo-colored based on their GABA response. A pseudo-colored image of the area is shown in Figure 5A. No organizational pattern based on the different maturity level of the neurons, as defined by the GABA response, was observed in the networks.

To assess how the heterogeneous network responded as a whole to increased GABA, calcium imaging and MEA recordings were studied on the network level. Synchronously active networks (five networks, 1702 neurons, Table 1, column 4) were used in this analysis because they were the only networks that showed stable network-wide activity. The global network activity was assessed as summed calcium fluctuations for calcium imaging and as firing rate of the whole MEA for one representative network as shown in Figure 5B. To compare the synchronous networks to each other, the network-level events, peaks of synchronous activity, were quantified with respect to their prominence from the baseline level of activity between peaks. The global activity peak prominence during calcium imaging at baseline and during GABA application is shown for each synchronous network (*n* = 5) in Figure 5C. The synchronous networks responded to GABA application in two different manners. The network-level activity was either strongly and significantly (*p* < 0.05) inhibited or not significantly (*p* > 0.05) affected, even though the networks contained cells excited by GABA. Furthermore, these two response groups differed in their baseline activity. The initial activity during synchrony was high (over 0.1 peak prominence) for networks strongly blocked by GABA and low (less than 0.05 peak prominence) for networks that barely responded to GABA. The change in interval between synchronous peaks of activity during GABA application was also analyzed but no clear change was observed.

The connection between the initial activity level and the GABA response was further studied on the single-neuron level by measuring the initial peak prominence and change in peak prominence during GABA application. The peak prominence associated with the initial activity is shown in Figure 5D. During GABA application, similar to the network-level responses, the individual neurons with a higher initial activity were more strongly inhibited than neurons with a low initial activity. The correlation between initial activity and strength of GABA effect for neurons within strongly or barely inhibited networks was −0.7273 (*p* < 0.0002) and −0.7175 (*p* < 0.000005), respectively. This seemed to be independent of the network they were from. To see if the composition of the neural networks was connected to the network-level response, the proportion of neurons with respect to their initial activity (Figure 5E) and strength of GABA inhibition (Figure 5F) was quantified for each network. The synchronous networks with higher initial activity contained a larger proportion of neurons with higher initial activity. This pattern was seen as an increased proportion of neurons with a peak prominence of 0.1–0.2. Likewise, the networks inhibited more strongly with GABA contained larger proportions of neurons with stronger GABA inhibition.

In summary, the network was observed to be a heterogeneous mixture of neurons with different responses to GABA. Furthermore, the synchronous networks were either highly active at baseline and strongly inhibited by GABA or less active during baseline and barely affected by GABA. Furthermore, on the single-cell level, initial activity was related to the GABA response in a network-independent manner. The proportion of neurons with high initial activity correlated with the network response to GABA.

### GABAergic Signaling in Synchronously Active Networks

The role of endogenous GABAergic signaling in synchronous network activity (five networks, 1702 neurons, Table 1, column 4) was studied by blocking GABA_A_ receptors with bicuculline (Bic). Responses to endogenous GABAergic activity blockade were analyzed from combined calcium imaging and MEA recordings. A representative calcium imaging and MEA recording from one network is shown in Figures 6A–C. Despite the presence of the GABA_A_ receptor blocker and the presence of GABA-responsive neurons, no disruption of the synchronous activity was observed. Thus, the synchronous activity is not completely dependent on GABAergic signaling.

The network-level response to blockade of endogenous GABAergic signaling was quantified to study if there was an effect. The global activity peak prominence during calcium imaging at baseline and during GABA_A_ receptor blockade is shown for each synchronous network (*n* = 5) in Figure 6D. The synchronous networks responded to GABAergic signaling blockade on the network level in two different manners. These two groups of networks were the same as those seen during GABA application and baseline. Endogenous GABAergic signaling blockade did not significantly (*p* > 0.05) inhibit the networks with a high initial activity but significantly significant (*p* < 0.05) increased activity in the networks with low initial activity (). The change in interval between synchronous peaks of activity during Bic application was analyzed but no clear change was observed.

The connection between the initial activity level and the response to GABAergic signaling blockade (with Bic) was further studied on the single-neuron level. The neuron responses as peak prominence with respect to initial activity are shown in Figure 6E. Unlike responses to GABA, the responses to blockade of GABAergic signaling did not depend on the initial activity of the neuron. The correlation between the initial activity and strength of GABA_A_ blockade for neurons within strongly or barely inhibited networks was 0.2022 (*p* = 0.293) and −0.2451 (*p* = 0.238), respectively. However, the single-neuron response was dependent on the behavior of the network it resided in. The proportions of neurons with respect to their response to GABAergic signaling blockade was quantified (Figure 6F). The distribution of single-neuron responses within the network seemed to determine the network-level response. Networks inhibited or stimulated by blockade of GABAergic signaling had larger proportions of neurons inhibited or stimulated by blockade of GABAergic signaling, respectively.

In summary, the blockade of endogenous GABAergic signaling did not disrupt the synchronous activity. However, the activity was decreased in networks with high and increased in networks with low initial activity. The response of the single neurons did not depend on their own initial activity but on the network in which they resided. Additionally, the distribution of single-neuron responses seemed to determine the network-level response to GABAergic signaling blockade.

### Role of Gap Junction-Mediated Signaling in Synchronously Active Networks

The role of gap junction-mediated signaling in synchronous network activity (five networks, 1702 neurons, Table 1, column 4) was studied by blocking gap junctions with CBX. The responses to application of the gap junction blocker during combined calcium imaging and MEA recording were analyzed. A representative calcium imaging and MEA recording from one network is shown in Figures 7A–C. The gap junction blocker used is known to have a slowly appearing effect. Thus, only the last 3–4 min of application were used for analysis. The gap junction blocker visibly decreased (4 of 5) or gradually abolished (1 of 5) the occurrence of synchronous peaks of activity. This was seen in both synchronous calcium rises (example recording in Figure 5A) and MEA recordings (example recording in Figure 5B). There was no clear change in the dynamics (rise time, decay time, peak width) of the single network-level peaks to gap junction blockade; however, the interval between peaks increased visibly.

To compare the network responses between synchronous networks, calcium imaging and MEA recordings were quantified on the network level, and peaks of network-level activity were detected from both data sets. The global activity peak interval and peak prominence during calcium imaging at baseline and during gap junction blockade are shown for each synchronous network (*n* = 5) in Figures 7D,E, respectively. Quantification of network activity peaks confirmed that the interval between peaks increased significantly (*p* < 0.01) in all synchronous networks with gap junction blockade Furthermore, the gap junction blocker caused a slight but significant (*p* < 0.05) reduction in peak prominence in all synchronous networks.

The synchronous networks were further studied on the single-neuron level by quantifying the inter-peak interval distribution for each network. Representative single-neuron peak interval histograms containing all neurons (*n* = 393) in one network during baseline and gap junction blockage are shown in Figure 7F. The network-level values seemed to correspond to a subpopulation within the distribution both before and after gap junction signaling blockade. To study if there was correlation between the network and subpopulation response, the distributions of single-neuron responses were analyzed for each network. Inter-peak interval change histograms for all synchronously active networks with their respective network changes are shown in Figure 7G. The gap junction blocker increased the interval between peaks in a subpopulation of neurons. Of the synchronous networks, 80% (4 of 5) contained a subpopulation of neurons that showed a similar inter-peak interval change compared to the change in the whole network. The distinct histogram peaks corresponding to the whole-network response suggests that there exists a gap junction-dependent population that regulates the activity of the whole network. We could not further identify this subpopulation based on physiological responses.

In summary, blockade of gap junction-mediated signaling was found to affect the synchronously active networks by decreasing the size and occurrence of the synchronous peak of activity. Furthermore, a subpopulation of neurons was observed to have an inter-peak interval change that was similar to that in the network response.

## Discussion

In this article, we describe the synchronous neuronal activity on a single-neuron and network level in hPSC-derived neural networks. Furthermore, we address two mechanisms generally responsible for the development of synchronous activity. In hPSC-derived neural cultures, synchronous activity was detected after 3 weeks of culture with calcium imaging and MEA recordings. The emergence of synchronous activity was found to associate with the decrease in excitatory GABA responses. Furthermore, the synchronous network activity did not depend on endogenous GABAergic signaling. Also, neurons with GABA responses varying from excitatory to inhibitory were found to act synchronously to give rise to the network activity. Large-scale analysis revealed that the network response was dependent on the responses at the single-neuron level. A subpopulation of neurons connected by gap junctions had a modulatory role in the synchronous network activity. Together, these results suggest that the earliest form of synchronous neuronal activity depends on gap junctions and a decrease in GABA excitability but not on endogenous GABAergic signaling.

hPSC-derived neurons form network-level activity patterns through their synchronization. This synchronization is dependent on decreased GABA excitability. Neurons with strong GABA induced depolarization did not participate in the synchronous activity. Furthermore, strong GABA depolarization of neurons was seen only in loosely synchronized and asynchronous networks. However, weakly GABA-depolarized neurons participated in the network activity in synchrony with strongly GABA-inhibited neurons. This observation is in line with previous studies where developing circuits in mouse PSC-derived networks (Illes et al., 2007, 2009, 2014; Risner-Janiczek et al., 2011) and rodent cortical cultures (Baltz et al., 2010) have been shown to also contain neurons inhibited by GABA. The very strong depolarizing GABA response might be due to the activation of higher affinity, extrasynaptic GABA_A_ receptors. These receptors are activated by ambient GABA as opposed to synaptic GABA and cause a very strong depolarization (Sipilä et al., 2005). The disappearance of the strongly GABA-depolarized neurons could mark the replacement of the extrasynaptic receptors with synaptic, lower affinity GABA receptors along with the maturation of network connectivity. The weak GABA depolarization, on the other hand, could be related to the dual role (Ben-Ari et al., 1997; Khazipov et al., 1997; Leinekugel et al., 1997; Lamsa et al., 2000; Cherubini and Ben-Ari, 2011) of GABA during network oscillations. The dual role arises from reversal of the Cl- gradient during activity and manifests as initial depolarization followed by inhibition. Such a switch would be possible with a lower but still depolarizing intracellular Cl- concentration. On the network level, we observed that the proportion of GABA-depolarized neurons was smaller in synchronous networks than in loosely synchronous and asynchronous networks. This difference is in line with previous studies that showed a decrease in the excitatory actions of GABA during network maturation in cortical cultures (Baltz et al., 2010) and *in vivo* (Ge et al., 2006). Furthermore, the addition of GABA inhibited network activity or had no effect. This result contrasts with the observations of excitatory GABA-driven network-level activity in *ex vivo* rat preparations (Leinekugel et al., 1997). The combination of the large-scale single-cell data with network activity showed that in our culture system, the switch from a depolarizing (excitatory) to inhibitory GABA response seemed to be a switch in individual neurons rather than in the whole network.

Here, the occurrence of synchronous network events did not depend on the GABAergic connections; however, the GABAergic connections did regulate network activity levels during synchronous events. The synchronous events occurred despite the presence of a GABA_A_ receptor antagonist. The use of Bic, however, does not allow the direct assessment of Bic insensitive GABA_C_ receptors or extrasynaptic receptors. Our observation is in line with previous reports of regular GABA-independent correlation in networks with functional GABA receptors from *ex vivo* rat (Garaschuk et al., 2000; Allene et al., 2008), *ex vivo* mouse cortex (Dupont et al., 2006; McCabe et al., 2006), *ex vivo* chick retina (Catsicas et al., 1998), embryonic rat cortical culture (Opitz et al., 2002; Baltz et al., 2010), and hPSC cultures (Kirwan et al., 2015). In contrast, GABAergic signaling has been shown to be required for correlated firing in *ex vivo* rat (Schwartz et al., 1998; Allene et al., 2008) and mouse (Aguiló et al., 1999) as well as embryonic rat cortical culture (Voigt et al., 2001). We observed that while the occurrence was not affected, the level of activity during the synchronous network events was decreased (similar to *ex vivo* mouse, Hunt et al., 2006) or increased in networks with high or low initial activity and strong or no response to externally applied GABA, respectively. The low initial activity, no response to externally applied GABA and stimulation by GABA_A_ receptor blockade could all be caused by tonic inhibition of the continuous activation of extrasynaptic GABA receptors (Glykys and Mody, 2007; Holter et al., 2007). On the other hand, the combination of high baseline activity, a strong inhibitory response to externally applied GABA and inhibition by GABA_A_ receptor blockade could in turn arise from activity-dependent depression via the GABA receptor (Chub and O’Donovan, 2001). Thus, the networks that respond differently to applied GABAergic modulators seem to contain differently acting GABAergic circuits that both regulate firing during network events. A shift in the behavior of developing GABAergic circuitry has previously been observed in *ex vivo* mouse cortex, where a GABA_A_ receptor blocker was initially inhibitory to network activity but became stimulatory during network maturation (Allene et al., 2008; Conhaim et al., 2011).

Gap junctions modulate the periodicity of the synchronous network activity by modulating the activity of a subpopulation of neurons. The gap junction blocker used (CBX) has been reported to have a broad range of nonspecific effects (Tovar et al., 2009). Here, the nonspecific effects were avoided by using a concentration <50 μM and observing the effect of CBX after 5 min of application. The gap junction blocker decreased the occurrence of synchronous events by increasing the interval between events. The change in the interval of the network events matched the change in the interval of the activity of a group of single neurons. Our results are in agreement with previous results obtained *in vivo* from the developing rat cortex (Yang et al., 2009), *ex vivo* fetal human tissue (Moore et al., 2014), and rat cortex (Yuste et al., 1995; Peinado, 2001; Molchanova et al., 2016), as well as *ex vivo* adult rat (Lamsa and Taira, 2003) and chick retina (Catsicas et al., 1998) where gap junction blockers decreased the occurrence of synchronous events. Thus, the gap junction-based mechanism for modulating the occurrence of synchronous network events can be argued to be retained in several biological models of developing neural networks. In contrast, the results obtained in mouse cortex *ex vivo* show that gap junction blockade completely blocks synchronous activity (Dupont et al., 2006; Hunt et al., 2006; Sun and Luhmann, 2007). Thus, this difference may suggest that the developing neural networks in mice may differ from those in rats, chicks and, ultimately, humans in this aspect.

Developing neural networks are affected by their constituent neural cells, and the formation of network functionality has been suggested to be impaired in cultured neural networks that lack astrocytes (Kuijlaars et al., 2016). The neural networks studied here have been shown to be primarily composed of neurons with a minor astrocyte population (Lappalainen et al., 2010). In this study, network activity was measured via functionality of its neuronal component, recorded by calcium imaging and MEAs. Similar to our previous studies (Heikkilä et al., 2009), synchronous activity was observed in MEA recordings from the hPSC-derived neural networks. This synchronous activity is a hallmark of developing neural networks and is a shared feature between *in vitro* (van Pelt et al., 2005; Illes et al., 2014) and *in vivo* (Garaschuk et al., 2000; Khazipov and Luhmann, 2006; Moore et al., 2011) networks, suggesting that developing neural networks can be accurately modeled with cell cultures. A difference between the developing networks *in vitro* and *in vivo* is the lack of the laminar structure in the former. However, a special network structure (i.e., cortical lamination) has previously been shown to be unnecessary for the formation of functional networks with rhythmic synchronized activity *in vivo* (Simmons and Pearlman, 1982), or *ex vivo* (Opitz et al., 2002). Thus, the observations from this study add to the growing body of evidence that the development of early network functionality in neuronal networks is not dependent on orderly structure. In conclusion, the described culture system and the observed network phenomena are a useful tool for studying the development of synchronous activity in developing neural circuits of human origin and the mechanisms behind the emergence of said activity. Studies performed with networks containing neurons starting from their most immature stage are important as they capture the mechanisms and spontaneous activity specific to a restricted period of development, not emerging in the adult brain (Blankenship and Feller, 2010; Momose-Sato and Sato, 2013). Furthermore, transient cell populations, some of which differ between primates and rodents (Hill and Walsh, 2005; Rakic, 2009), emerge and disappear during early network formation, laying the groundwork for further network development (Luskin and Shatz, 1985). Such populations and their contribution to the network activity development can only be observed in human-derived immature neural cell cultures.

### Summary

In conclusion, we showed that the single-neuron excitatory response to GABA decreases as neurons start to participate in synchronous network activity. As this change occurs, these neurons give up their individual activity pattern and jointly give rise to the network activity pattern. In hPSC-derived networks, this activity pattern is at least partially modulated by connections between neurons. Furthermore, we showed that gap junction-mediated connections modulate the interval between events, allowing network events to occur more frequently. In addition, GABAergic connections are not necessary for the occurrence of network events but act as limiters of activity during synchronous events. Together this control over the occurrence and level of activity during synchronous events allows the networks to control the activity of its single-neuron components. The control over activity in turn allows the immature neural networks to form an internally controlled activity pattern.

## Author Contributions

ME-LM designed the overall study, performed the experiments, designed, programmed and tested the analysis used, interpreted the data for the work, as well as wrote the manuscript. LY-O and SN supervised the study and gave valuable comments on the manuscript. All authors contributed to manuscript revision, read and approved the submitted version.

## Conflict of Interest Statement

The authors declare that the research was conducted in the absence of any commercial or financial relationships that could be construed as a potential conflict of interest.
