# Supplementary material for: GABA and Gap Junctions in the Development of Synchronized Activity in Human Pluripotent Stem Cell-Derived Neural Networks
Source: Front Cell Neurosci. 2018 Mar 6;12:56. doi: 10.3389/fncel.2018.00056 (PMC5845705; doi:10.3389/fncel.2018.00056)
Supplement: Supplementary file 1 [file Data_Sheet_1.docx]

Supplementary Material

GABA and gap junctions in the development of synchronized activity in human pluripotent stem cell-derived neural networks

Meeri Eeva-Liisa Mäkinen*, Laura Ylä-Outinen, Susanna Narkilahti

*** Correspondence:** Corresponding Author: meeri.makinen@uta.fi

# Supplementary Figures and Tables

## Supplementary Figures

**
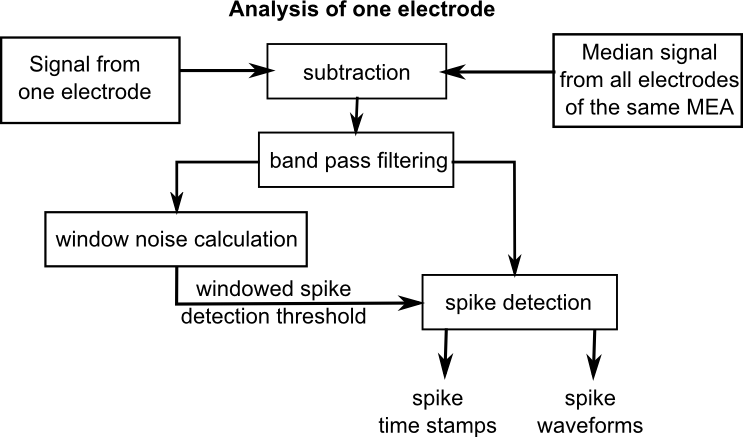
**

**Supplementary Figure 1.** The analysis process for one electrode during the MEA analysis. The median signal calculated across all electrodes is subtracted from the electrode signal. This subtraction is followed by filtering. The filtered signal is divided into windows, and the noise of each window is used to set the spike detection threshold for that window. The spike detection threshold is then used to detect spikes in the filtered signal. The output results are spike timestamps and cut-out waveforms.

**
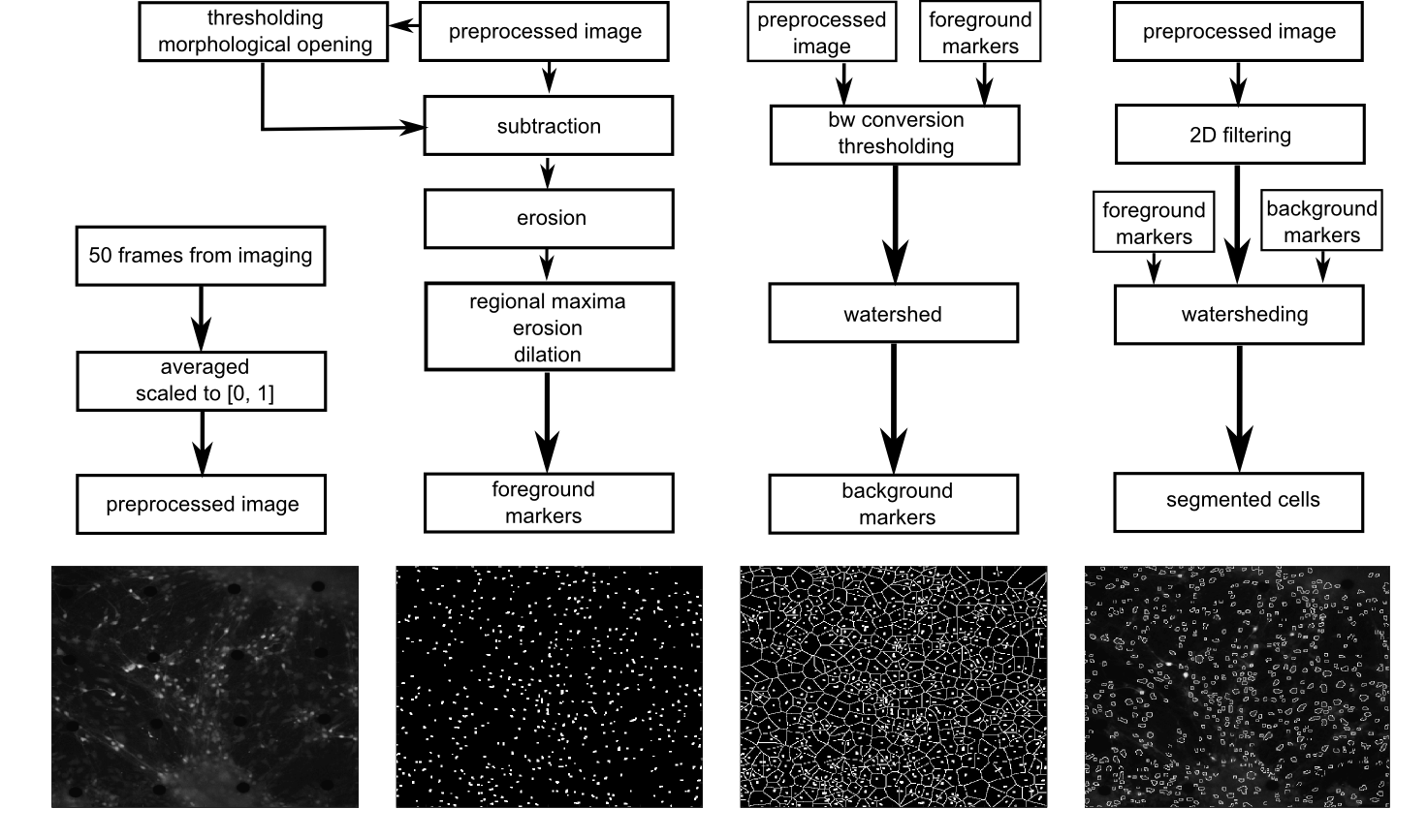
**

**Supplementary Figure 2.** The analysis process for a set of 50 subsequent images from the calcium imaging analysis. The first column depicts the preprocessing of the 50 frames into an averaged image. The second column depicts the analysis process for the generation of foreground markers from the preprocessed image. The third column depicts the analysis process for generating the background markers. The fourth column depicts the final segmentation analysis process from the preprocessed image, foreground and background markers. The bottom row contains images from different parts of the analysis of the same set of images. From left to right: preprocessed image, foreground markers, background markers and the final segmentation result.

**
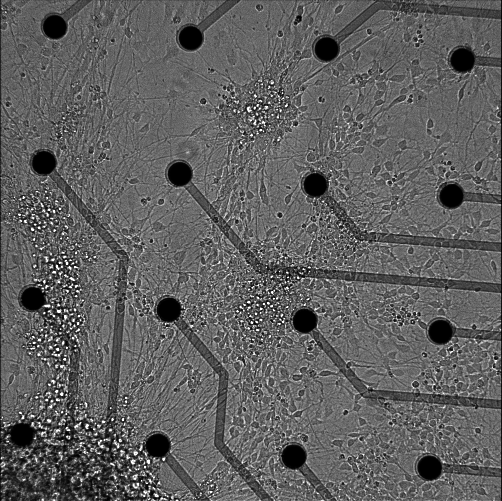
**

**Supplementary Figure 3.** Two-week-old hPSC-derived neural culture on top of a thin MEA (MEA dishes with 180 µm thick recording area) a few minutes prior to calcium imaging. The distance between two electrodes is 200 µm.

**
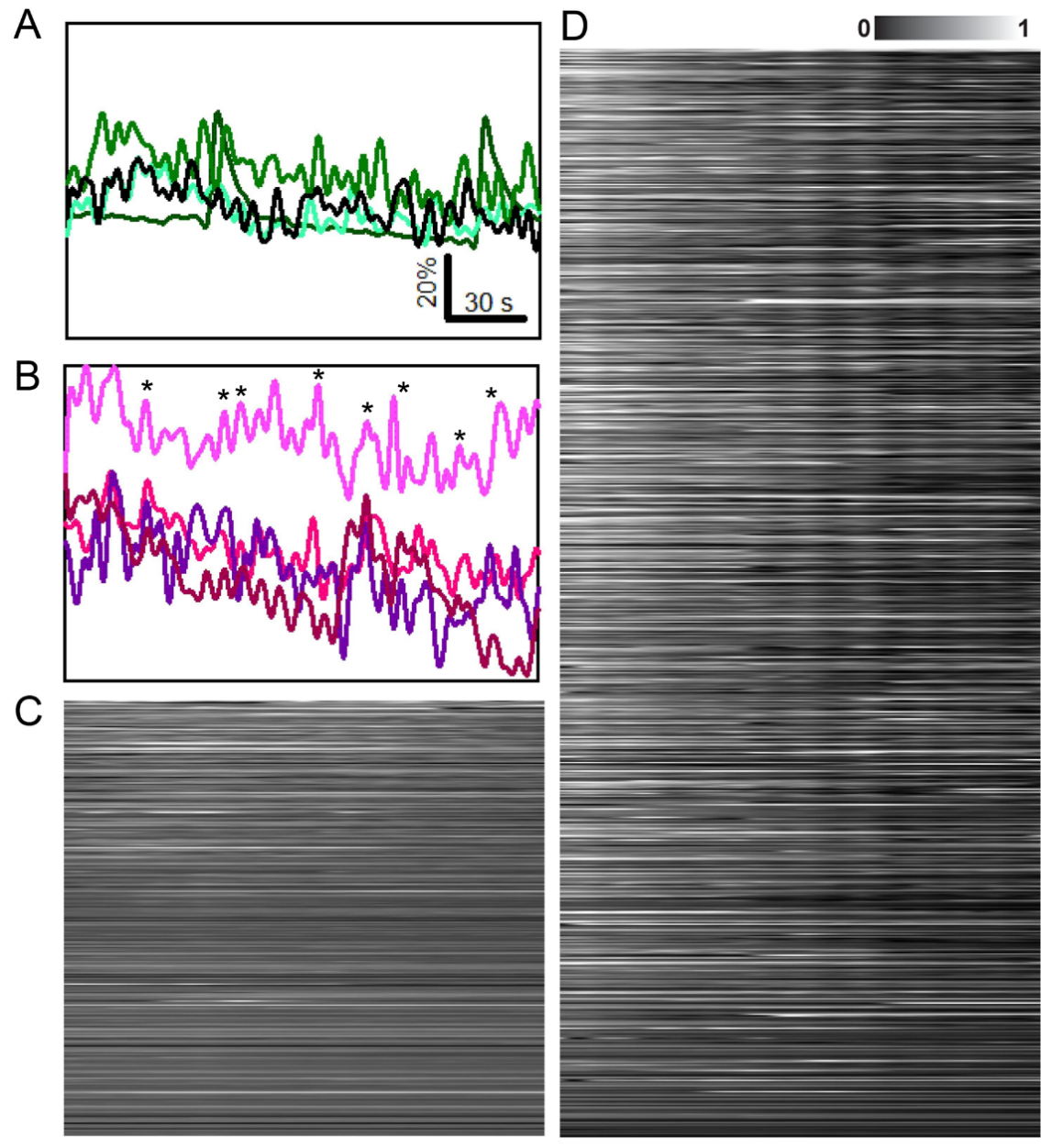
**

**Supplementary Figure 4.** **(A)** Representative single-neuron intracellular calcium level traces from 4 asynchronously active neurons. Neurons are selected from the recording shown in (C). Traces show the normalized fluorescent calcium dye intensity (y-axis) over time (x-axis). **(B)** Representative single-neuron intracellular calcium level traces from 4 loosely synchronously active neurons. Neurons are selected from the recording shown in (D). Traces show the normalized fluorescent calcium dye intensity (y-axis) over time (x-axis). Scale same as in (A). The asterisks mark the moments of synchronous activity. **(C)** Normalized (scalebar) raster plot of spontaneous intracellular Ca2+ fluctuations from all neurons (n = 403) in the field of view from one asynchronous network. Each row of pixels (y-axis) contains the normalized intensity changes during a 5-min (x-axis) recording from one neuron. **(D)** Normalized (scalebar) raster plot of spontaneous intracellular Ca2+ fluctuations from all neurons (n = 774) in the field of view from one loosely synchronous network. Each row of pixels (y-axis) contains the normalized intensity changes during a 5-min (x-axis) recording from one neuron.
